# A Review of Iodine Status of Women of Reproductive Age in the USA

**DOI:** 10.1007/s12011-018-1606-5

**Published:** 2019-01-07

**Authors:** Pallavi Panth, Gena Guerin, Nancy M. DiMarco

**Affiliations:** 1 Department of Nutrition and Food Sciences, College of Health Sciences, Texas Woman’s University, P.O. Box 425888, Denton, TX 76204-5876 USA; 2 Institute for Women’s Health, College of Health Sciences, Texas Woman’s University, Denton, TX 76204-5876 USA; 3 Department of Kinesiology, College of Health Sciences, Texas Woman’s University, Denton, TX 76204-5876 USA

**Keywords:** Iodine, Iodine deficiency, Iodine status, Women of reproductive age, Thyroid, Urinary iodine

## Abstract

Iodine, an essential micronutrient, is required to produce thyroid hormones. Iodine deficiency disorders (IDD) comprise a range of adverse maternal and fetal outcomes, with the most significant irreversible effect resulting from neurodevelopmental deficits in fetal brain caused by deficient iodine status during early pregnancy. The objective of this scoping review was to summarize the studies that assessed iodine status of women of reproductive age in the USA. A systematic review of literature using the PRISMA Extension for Scoping Reviews (PRISMA-ScR) statement was conducted. PubMed, Medline, CINAHL, EMBASE, EBSCOHost, Cochrane, ERIC, Google Scholar, and Web of Science databases were searched, 1652 records were identified. One thousand six hundred forty-one records that did not satisfy the inclusion/exclusion criteria and quality review were excluded, and 11 peer-reviewed articles were determined to be eligible for this scoping review. Despite the USA being considered iodine sufficient for the general population, the US dietary iodine intakes have decreased drastically since the 1970s, with iodine deficiency reemerging in vulnerable groups such as women of reproductive age. Although data to conduct a scoping review of iodine status among women of reproductive age in the USA was scarce, majority of the articles reviewed demonstrate emergent iodine deficiency in this population of women of reproductive age, indicating alarm for a public health concern needing immediate attention.

## Introduction

Iodine, an essential micronutrient, is found in every tissue in the body. The only known function of iodine is its role in the production of thyroid hormones, thyroxine, T_4_, and triiodothyronine, T_3_, although it may also act as an anti-oxidant, anti-inflammatory, apoptotic, antiviral, and antibacterial agent [1–3]. Thyroid hormones play essential roles in regulating energy homeostasis by modifying basal metabolic rate and thermogenesis [4]. It is imperative to know how much iodine is necessary for optimal health to understand iodine sufficiency. The recommended dietary allowance (RDA) for iodine is 150 μg/day for healthy adults [5]. The recommended method to assess iodine adequacy from the diet is by analyzing urine iodine concentration (UIC) as 90% of dietary iodine is excreted in the urine [6]. For this reason, UIC is an excellent marker of recent iodine intake at a population level. UIC is measured in spot urine samples from a representative sample of women and expressed as a median due to the high variability of spot urine samples [7]. The World Health Organization (WHO) determined the UIC of 100–199 μg/L indicates adequate iodine nutrition status for adults [6]. In non-pregnant women, median UIC between 100 and 299 μg/L defines a population which has no iodine deficiency. In addition, not more than 20% of samples should have a UIC below 50 μg/L. This UIC of 100–199 μg/L corresponds approximately to a daily iodine intake of 150 μg/day for adults, which includes non-pregnant women [6]. Therefore, the Institutes of Medicine (IOM) RDA of iodine of 150 μg/day for adults corresponds with 70–80% of daily iodine intake [5, 6, 8]. It is crucial to remember at this juncture, since UIC is evaluated as a median, by definition, when the median UIC is 100 μg/L, at least 50% of the samples will have a UIC lower than 100 μg/L [5, 6, 9]. The RDA for iodine is 220 μg/day for pregnant and 290 μg/day for lactating women [5, 6]. Although UIC 100–199 μg/L for evaluation of iodine sufficiency applies to adults, it does not apply to pregnant and lactating women. For pregnant women, median UIC of < 150 μg/L indicates insufficiency, and 150–249 μg/L indicates adequate iodine nutrition status. For lactating women, median UIC 100 μg/L can be used to define adequate iodine nutrition status, but no other categories of iodine intake are defined. Lactating women have the same iodine requirement as pregnant women; however, the median UIC is lower because iodine is secreted in breast milk [5, 6]. According to the Iodine Global Network (IGN) Global Scorecard of Iodine Nutrition 2017, pregnant women in the USA have insufficient iodine intake [10]. Median UIC has shown a decreasing trend from 320 μg/L in 1974 to 144 μg/L in 2010, suggesting a more than 50% reduction in dietary intake [11, 12]. Thirty-six percent of US women had UIC < 100 μg/L, and 16% of women had UIC < 50 μg/L, signifying that 2.2 million women have low or deficient iodine intakes [13]. The downward trend from iodine sufficiency in the early 1970s to iodine deficiency has plateaued at a level significantly below sufficiency for women of reproductive age in the USA [12, 13].

Iodine status differs in men and women, and iodine deficiency has more severe consequences for women as it will affect future generations [14]. The iodine requirement during pregnancy is significantly increased because of an increase in maternal T_4_ production to maintain normal maternal thyroid function and transfer thyroid hormone to the fetus early in the first trimester, before the fetal thyroid is functioning, iodine transfer to the fetus, particularly in later gestation, and an increase in renal iodine clearance throughout pregnancy [15]. Women should ideally be provided with iodine intake of at least 150 μg/day for a long period *before conception* to ensure plentiful intrathyroidal iodine stores and adequate iodine supply during pregnancy [9, 14].

Iodine intake in the US diet has steadily decreased over the past few decades. Women of reproductive age who consume similar iodine-deficient diets are especially vulnerable to maternal and fetal effects of iodine deficiency. These women may become pregnant during these reproductive years and will be iodine deficient at the beginning of pregnancy and, without supplementation, throughout the pregnancy, and into lactation. Since this is a critical time for development in the life of the fetus and infant, this may be catastrophic. Iodine deficiency has multiple adverse effects on growth and development of the fetus and the resulting conditions are collectively termed iodine deficiency disorders (IDD) [1, 2]. Iodine deficiency in pregnancy impairs the neurological development of the fetus, as thyroid hormone is required for normal neuronal migration and myelination of the brain during fetal and early postnatal life [16]. Hypothyroxinemia due to iodine deficiency in the mother resulting in inadequate thyroid hormone release to the fetus during these critical periods causes irreversible brain damage, with mental retardation [16]. Iodine deficiency is the leading cause of preventable mental retardation in the world [1, 10, 16].

Although iodine deficiency is historically considered to be a problem of developing countries, it can affect both developing and industrialized nations. More importantly, iodine deficiency has recently begun to reappear in some nations that were previously iodine sufficient. Recent studies have suggested that vulnerable UK populations, such as women of reproductive age, pregnant, and lactating women might again be iodine deficient due to a decrease in UK milk consumption [17, 18]. Similar reemergence of iodine deficiency has also been observed in European nations such as Finland, Italy, Hungary, France, Belgium, and Spain [19]. Australia is another example of an industrialized nation that was iodine sufficient for decades until changes in dairy industry practices inadvertently reduced the iodine content of dairy products, contributing to iodine deficiency [20, 21]. The trend of reemergence of iodine deficiency among vulnerable populations such as reproductive age women in the USA appears to mirror the trends in these other industrialized nations.

Public awareness of the importance of iodine consumption in women of reproductive age, especially in the prenatal period and the first trimester of pregnancy is severely lacking [22]. WHO and the American Thyroid Association (ATA) recommend iodine supplementation of all pregnant women in the range of at least 250 μg/day where iodine intake may be insufficient, but it has not been widely adopted [23, 24]. In 2017, the ATA added that women who are planning pregnancy should begin taking a supplement with iodine 3 months in advance of the planned pregnancy [25]. Over 75% of obstetricians and midwives do not recommend iodine supplementation to patients planning to become pregnant, during pregnancy, or lactation despite the deleterious effects of iodine deficiency in women of reproductive age [26]. Also, the fact that as many as 50% of prenatal vitamins currently available on the market do not contain iodine at all and those that do may have inconsistent labeling is troubling [27, 28].

Although some studies have looked at the iodine status of school-aged children, pregnant, and lactating women, few have focused on the iodine status of women of reproductive age in the USA as the USA is generally considered to be an iodine-sufficient nation. The purpose of this scoping review is to summarize the iodine status of women of reproductive age in the USA and address possible causes of iodine deficiency in this subset of the US population in the discussion. It is possible that a simple correction of iodine status may help prevent some of the hidden or overt adverse outcomes associated with iodine deficiency which may currently be going unnoticed in women of reproductive age in the USA.

## Materials and Methods

### Search Strategy and Data Collection

**Fig. 1:**
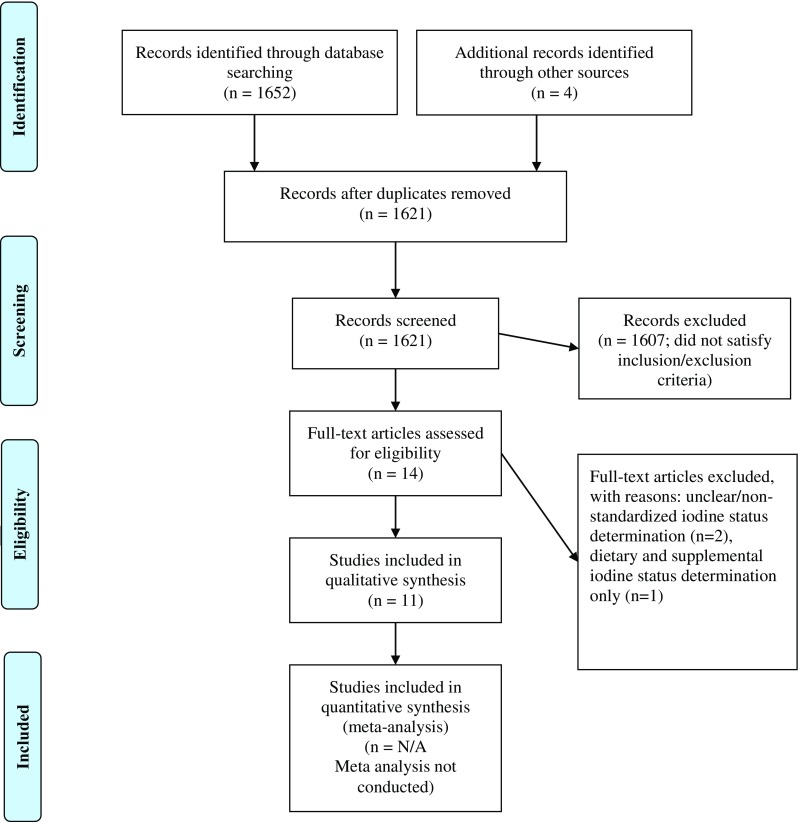
Study selection PRISMA flow chart

This study is a scoping review, so an ethical statement is not required. This qualitative scoping review is reported in accordance with the PRISMA Extension for Scoping Reviews (PRISMA-ScR) statement [29]. The study selection process is documented in Fig. 1. A systematic literature search was performed in PubMed, Medline, CINAHL, EMBASE, EBSCOHost, Cochrane, ERIC, Google Scholar, and Web of Science databases to retrieve all studies conducted in the USA that may have assessed iodine status of women of reproductive age in the USA. Both text word search and keyword search using MeSH definitions were used. Search terms and combinations of the MeSH terms included iodine, urinary iodine, iodine deficiency, iodine status, pregnancy, maternal, women of-reproductive age, reproductive age women, prenatal, perinatal, thyroid hormone, lactation, fertile age, childbearing age, systematic review, United States. Study reports from book chapters, master’s theses, and doctoral dissertations were investigated in the ProQuest database, and references in review papers and non-indexed journals were also searched by the authors. Duplicate publications were checked and eliminated, and if necessary, corresponding authors were contacted via email for additional information. The start period of studies being conducted was not limited to current articles as the authors wanted to retrieve all studies conducted in the USA that may have assessed iodine status among this population in the USA. Therefore, a search for articles published from 1970 through April 2018 was conducted. The year 1970 was also selected as the start point for the search as the first National Health and Nutrition Examination Survey (NHANES) study was conducted in 1971. NHANES is a program of studies designed to assess the health and nutritional status of adults and children in the USA and is generally considered to be the more comprehensive database tracking iodine nutrition status of all subsets of the US population [30]. All research studies reporting NHANES data for women of reproductive age in the USA have been included in this review. A total of 1652 records were identified initially. After duplicates were removed, a total of 1621 citations were identified from searches of electronic databases and review article references. Based on the title and the abstract, and inclusion/exclusion criteria, 1607 were excluded, with 14 full-text articles to be retrieved and assessed for eligibility. Of these, 3 were excluded for the following reasons: 2 did not use standardized iodine status determinations, and one assessed only dietary iodine intake via supplement use. The remaining 11 studies were considered eligible for this review.

A meta-analysis was not attempted as the number of research studies assessing the iodine nutrition status of women of reproductive age in the USA was very small, and majority of studies primarily analyzed the NHANES database to report iodine status of the US population, accounting for repetition of results across the articles that analyzed iodine status in women of reproductive age in the USA. Hence, the results of this study are being reported as a scoping review.

The main outcome measured in this scoping review was iodine status in women of reproductive age in the USA. This scoping review was conducted to systematically map the research done in this area, as well as to identify any existing gaps in knowledge, and to potentially discuss causes and the significance of iodine deficiency in this subset of the US population. The following research question was formulated: What is known from the literature about the iodine status of women of reproductive age in the USA since 1970 to present time?

### Inclusion Criteria

This review includes 11 peer-reviewed journal articles that met the following inclusion criteria: the language in which the article was published was in English, the main purpose of the article was the assessment of iodine nutrition status of individuals residing in the USA, primary studies were conducted in the USA, analysis of iodine status of women of reproductive age 18–44 years of age, including non-pregnant, pregnant, and lactating women, studies documenting standardized WHO/IOM guidelines for iodine sufficiency, markers for iodine status determination such as 24-h UIC, spot UIC or iodine: creatinine ratio assessments. Normal reference range for adequate iodine nutrition as evaluated by UIC for women of reproductive age and pregnant women was 150–249 μg/L [6].

### Exclusion Criteria

Animal studies, review articles, case studies, case reports, studies investigating the iodine status of infants, children, and older adults, studies investigating iodine status in individuals with thyroid diseases and/or other chronic diseases, studies investigating radioiodine treatment, and duplicate articles were excluded from this review. The rationale for the inclusion/exclusion criteria was to identify all possible peer-reviewed journal articles that may have reported the iodine status of women of reproductive age in the USA for the purposes of this scoping review.

### Quality Assessment of Reviewed Articles

Two of the authors (P.P and N.M.D) worked independently by utilizing the Academy of Nutrition and Dietetics Evidence Analysis Library Worksheet to critically assess each included article for methodologic quality. Each study was evaluated based on appropriateness of study design and the quality of how the study was conducted by using the Academy’s risk of bias tool called the Quality Criteria Checklist (QCC) [31]. Articles were then given a grade to assess the strength of the evidence as follows: grade I, good; grade II, fair; grade III, limited; grade IV, expert opinion only; and grade V, non-assignable. All articles deemed eligible for this review were assessed as grades I–III (*n* = 11/12) and were included in this review. Data was extracted from the eligible articles by one reviewer and responses verified by another.

## Results

### Studies Reviewed

A total of 1652 records were identified in the initial search including MeSH terms and combinations of the MeSH terms from the databases reported in the Methods. Figure 1 documents the study selection process using the PRISMA-ScR statement. Toward the end of the search and eligibility determination process, 11 studies published between 1970 to April 2018 were found to be eligible for this scoping review based on the inclusion/exclusion criteria listed in the “Methods”. Ten studies were cross-sectional studies, and one study was a randomized controlled trial. NHANES data was the primary reported data for iodine status of the US population, including the population of interest, women of reproductive age. The characteristics of the study participants, methods of iodine status determination, and outcomes for the 11 studies selected for this scoping review are reported in Table 1.

**Table 1:** Characteristics of subjects, methods and results from studies included in this review

Study	Data	Subjects	Design	Methods	Results		
					Overall Median UIC	Pregnant Median UIC	Non-pregnant Median UIC
Caldwell et al. (2005) [32]	NHANES 1988-1994,	Women 15-44 yrs, n=5405, 348 pregnant, 5057 non- pregnant	Cross-sectional	Urine iodine spot test, urinary creatinine	128 μg/L, 36.1% <100 μg/L	141 μg/L, 28.5%<100 μg/L	127 μg/L, 36.5%<100μg/L
	2001-2002	n=679, 126 pregnant, 132 non- pregnant			132.5 μg/L, 38% <100 μg/L	172.6 μg/L, 37.7%<100 μg/L	132 μg/L, 38%<100μg/L
Caldwell et al. (2008) [33]	NHANES 2003-2004	Women 15-44 yrs	Cross-sectional	Urine iodine spot test, urinary creatinine	139 μg/L, 37.2% <100 μg/L	181 μg/L	131 μg/L
Caldwell et al. (2011) [27]	NHANES combined 2005-2006 2007-2008	Subgroup women 15-44 yrs	Cross-sectional	Urine iodine spot test, urinary creatinine	76.2 million women with UI < 50 μg/L	125 μg/L 56.9% of all pregnant women UI< 150 μg/L (n= 184), 1.9 million pregnant women Trimester I=182 μg/L, Trimester II=154.6 μg/L, Trimester III=135.9 μg/L	130 μg/L 29.5 million non-pregnant women <WHO recommendations, 7.6 million women ~14.8% non-pregnant women <50 μg/L
Caldwell et al. (2013) [12]	NHANES 2005-10	2233 Women 15-44 yrs 206 pregnant, 2027 non-pregnant,	Cross-sectional	Urine iodine spot test, urinary creatinine	124 μg/L	42 females, Trimester I= 109 μg/L 70 females, Trimester II= 128 μg/L 64 females, Trimester III= 172 μg/L	133 μg/L
	NCS Vanguard Study 2009-2010	501 pregnant women (NCS) Trimester III				167 μg/L	
Hollowell et al. (1998) [39]	NHANES I, III	Women 18-44 yrs	Cross-sectional	Fasted, urine iodine spot test, urinary creatinine	Percentage of 15-44 yrs women with UIC <50 μg/dL increased 3.8 times between studies	UIC <50 μg/dL increased 6.9 times	
Hollowell and Haddow (2007) [34]	NHANES I 1971-1974	5279 women, 15-44 yrs, 208 pregnant,	Cross-sectional	Urine iodine spot test, urinary creatinine	294 μg/L	327 μg/L	293 μg/L
	NHANES III 1988-1994	5405 women, 15-44 yrs, 312 pregnant	Cross-sectional		128 μg/L	141 μg/L	127 μg/L
Lee et al. (2016) [37]	NHANES	12779, 20> yrs, 51% women	Cross-sectional	Urine iodine spot test	20-39 yrs, 129.8 μg/L 40-59 yrs, 123.6 μg/L		
	2001-2004	20-39 yrs 40-59 yrs			122.7 μg/L 122.1 μg/L		
	2005-2008	20-39 yrs 40-59 yrs			135.1 μg/L 126.6 μg/L		
	2009-2012	20-39 yrs 40-59 yrs			119.5 μg/L 117.1 μg/L		
Mills et al. (2018) [35]	LIFE Study 2005-2009	501 women, 18-40 yrs, non-pregnant	Cross-sectional	Spot urine for iodine and creatinine	112.8 μg/L 102, mildly deficient UIC 50-99 μg/L, 97, moderately deficient UIC 20-49 μg/L, 8, severely deficient UIC <20 μg/L	97.2 μg/L	113.6 μg/L
Pan et al. (2013) [38]	NHANES 2001-2010	3935 women 15-44 yrs, 415 pregnant women	Cross-sectional	Urine iodine spot test, urinary creatinine	Overall median UI for all females, 141.8 μg/L Women 15-44 yrs median UI=131.1 μg/L,	Median UI=145 μg/L Pregnant women >35 yrs higher median UI than pregnant women <35 yrs	
Pessah-Pollack et al. (2014) [82]	New York City Health Clinic	182 pregnant women	Randomized Controlled Trial 150 μg KI, supplemented vs non-supplemented	UIC spot test	Overall median UIC 152 μg/L, supplemented group median UIC 169.8 μg/L, non-supplemented group median UIC 128.4 μg/L, non-supplemented group 38.9% risk for IDD vs 22.8% of supplemented group		
Perrine et al. (2010) [36]	NHANES 2001-2006	326 pregnant, 15-44 yrs 53 lactating, 1437 non- pregnant/non- lactating	Cross-sectional	Urine iodine spot test		Pregnant, median UIC = 153μg/L, lactating = 115μg/L	Non-pregnant, non-lactating UIC= 130 μg/L

Of 11 reviewed studies, seven included data from non-pregnant women ages 15–44 years indicating that the US population is at risk for iodine deficiency per urine spot testing [12, 27, 32, 33, 34, 35, 36]. Median UIC in pregnant women of reproductive age showed a decreasing trend through the years, from 294 μg/L in NHANES I to 128 μg/L in NHANES III; median UIC of women non-pregnant women decreased from 293 to 127 μg/L during the same period [34]. Most recent data from the LIFE study [35] indicates a dramatic reduction in median UIC levels in pregnant women to 97.2 μg/L, and non-pregnant women to 113.6 μg/L, indicating a significant cause for concern, as these values fall well below the recommended WHO range for median UIC in pregnant females. Seventeen percent of the US adult population were iodine deficient, with a median UIC < 150 μg/L, median UIC for all females was 134 μg/L, and median UIC for women of reproductive age was 124 μg/L [12]. Thirty-six percent of women had UIC < 100 μg/L, and 16% of women had UIC < 50 μg/L [12]. In an NHANES study combining the data from 2005 to 2006 and 2007 to 2008, the median UIC was 125 μg/L for pregnant women and 130 μg/L for non-pregnant women. 1.9 million pregnant women and 29.5 million non-pregnant women were found to have UIC below WHO recommendations of 100 μg/L, and more significantly, 7.6 million women has UIC < 50 μg/L [27]. The first, second, and third trimesters were divided into median UIC, trimester I = 182 μg/L, trimester II = 154.6 μg/L, and trimester III = 135.9 μg/L [27]. One of the studies indicated that women 20–39 years had the lowest median UIC of 119.5 μg/L indicating deficient iodine status in this sub-group of reproductive age women [37]. Another study determined that percentage of women of reproductive age, 18–44 years, and pregnant women with iodine deficiency (< 50 μg/L) increased by 6.9 times in the period between the 1971–1974 and 1988–1994 NHANES data collection [39]. Of 11 reviewed studies, seven studies indicated that pregnant women in age group 15–44 years are at risk or have deficient iodine status, and the numbers of pregnant women assessed as being deficient have increased since NHANES 1971–74 [34, 35, 38, 40].

Women of reproductive age in the USA are therefore iodine deficient according to the analysis of NHANES data. Since the original NHANES I (1971–1974) study, non-pregnant women have been below the WHO guidelines for UIC indicating inadequate iodine nutrition. The period between NHANES I and NHANES III captured the most significant decline in iodine levels in about 20 years [27, 34]. Analyzing data from 2001 through 2010 continued to document iodine deficiency in women of reproductive age. Median UIC may be stabilized; however, they are stabilized at deficient levels. The most plausible reason for this drop in UIC is that women of reproductive age may not have sufficient dietary iodine intakes to support thyroid and metabolic health and the future neurological health of the developing fetus. Of all the dietary sources of iodine, dairy intake was found to be the leading indicator of iodine consumption and thereby iodine status [37]. Between 2001 and 2012, the percentage of women with UIC < 50 μg/L or severe iodine deficiency increased from 11.6 to 13.2% [37]. Women who consume dairy were found to have a lower risk of iodine deficiency than those who did not. Iodine supplementation also correlated with adequate iodine status in women of reproductive age [41]. It then becomes imperative that women have sufficient dietary iodine intake or supplement with iodine, especially in the first and second trimesters, to allow for thyroid hormone production for fetal brain development. Women had lower UIC than men in every NHANES data collection since 1971–1974.

## Discussion

### Iodine Status Assessments: Considerations and Limitations

Iodine intake is a crucial determinant of iodine status but is difficult to assess. Urinary iodine concentration (UIC) is the method most commonly used to assess dietary iodine intake. UIC has been measured using spot UIC by the Centers for Disease Control (CDC) National Health and Nutrition Examination Survey (NHANES) from 1972 to the present [30]. NHANES data has consistently been the only assessment utilized for iodine sufficiency in the USA. This method provides information on iodine status based on one point in time and has limitations to its validity [42]. For population-based studies, only spot UIC collection is logistically possible, but the data may not be reflective of an individual’s iodine status; thus, when extrapolated to a population, the results should be interpreted with caution. The determination of UIC provides little information on the long-term iodine status of an individual as spot samples are a one-time glance at the concentration of excreted iodine [6, 7]. This method has inherent problems as it does not take into consideration factors affecting iodine excretion. Possibly, recent consumption of foods with high iodine content, high fluid volume, and goitrogens could account for falsely high iodine excretion. Iodine excretion may also correlate with estrogen variability of the menstrual cycle [43]. Therefore, the day of UIC collection may influence iodine excretion up or down. Another method to assess iodine levels is by 24-h UIC, which represents an individual’s single day excretion of iodine. Although this is a more accurate estimate of dietary iodine intakes, it is not feasible for a population study. The study by König, et al. reported that it would take ten spot UIC tests to have any degree of accuracy, and even one 24-h sample has only 20% reliability estimating iodine status [42]. Although spot UIC and 24-h UIC may be valid tools to evaluate population iodine status, neither can be used to critically evaluate an individual’s iodine nutrition status. Urinary iodine per gram creatinine ratio (UIC/Cr) of a single voided urine sample is another method to quantify iodine in individuals. It was thought to be the best way to compensate for variability of urine volumes as daily creatinine excretion was assumed to be relatively constant. However, limitations of assessing UIC/Cr include due to day to day variation in UCr excretion and low excretion rates in populations with inadequate protein intake [44].

For women of reproductive age, achieving iodine sufficiency should be a priority. The iodine loading protocol developed by Abraham et al. may be another option to determine individual iodine status [45]. The basis for the test is to examine how much iodine is excreted compared to the load consumed, with the reasoning being that the body will clear whatever amount of iodine/iodide is not absorbed from the loading dose of 50 mg. Excretion of 45 mg, or about 90%, means the individual is iodine sufficient [46]. This method of iodine testing, although being used by independent labs across the USA, is not a validated tool to evaluate individual iodine nutrition status. It may also be difficult to extrapolate the results of this test to the WHO/IOM criteria for the definition of iodine deficiency in populations.

Measures of thyroid function also indicate iodine status [47]. If serum TSH and T_4_ are within normal limits, individuals are assumed to be iodine sufficient, since a decrease in T_4_ and increase in TSH only occurs when iodine deficiency is severe [48]. The American Thyroid Association states there are 20 million Americans with thyroid disorders and 13 million are undiagnosed with a majority of the diagnosed and undiagnosed population comprised of women, especially women of reproductive age [23]. These assumptions are based on the current evaluation of TSH levels and depending upon a “normal” range of values, and therefore accounts for the significant discrepancy in numbers. Nevertheless, this would mean ~ 20% of our population is at risk for thyroid disorders, a number not unlike the population observed in the Goiter Belt before salt iodization in the 1920s. According to the Mayo Clinic, accurate thyroid function tests are available to diagnose hypothyroidism, and treatment of hypothyroidism with synthetic thyroid hormone is usually simple, safe, and effective [49]. However, iodine deficiency is not a consideration when diagnosing hypothyroidism, even though iodine is essential to produce thyroid hormones, and iodine deficiency is one of the leading causes of hypothyroidism. Conversely, taking in too much iodine can also cause hypothyroidism [23, 49]. Therefore, we have a problem that we do not take in enough iodine because we are fearful of causing thyroid dysfunction, but because of iodine deficiency, we have an epidemic of thyroid disorders that is currently only being treated by taking an over-prescribed drug that eliminates healthy thyroid functioning. It might be simpler to mandate iodization of salt or improve iodine intake from diet or supplements rather than creating a levothyroxine-dependent hypothyroid population.

### Dietary Iodine Deficiency

Historically, iodine deficiency in the USA was a matter of geographic location. Iodine is found in varying quantities in the oceans and on land masses. The “Goiter Belt” included states from the Pacific Northwest, the northern Rocky Mountains, to the Midwest, Great Lakes, and Appalachia. Areas prone to flooding and mountainous terrain are consistently iodine depleted. Food sources from these areas lack iodine, and therefore, goiter was prevalent. Severe iodine deficiency manifests as a goiter. In the 1820s, J. F. Coindet, a Swiss physician, recognized iodine for its ability to reduce goiter size. By 1920, David Marine, M. D., from Cleveland, OH, in the Goiter Belt region, brought iodine treatment to the forefront in the USA. Dr. Marine first studied iodine on animals and then on school girls with goiter. Sodium iodide supplementation among adolescent girls reduced thyroid enlargement, a result of iodine deficiency, from 21 to 0.2%. This study is what led to the iodization of salt in the USA. Goiters, a common problem in the early twentieth century and the result of iodine deficiency, became a thing of the past with salt iodization. Salt iodization programs, including bread fortification, began as a result of the work of these men. Due to the iodization of salt in the 1920s, iodine deficiency and the symptoms associated with it were markedly reduced [50].

Iodine deficiency during fetal and infant developmental stages may cause mental retardation, autism, developmental delays, cretinism, goiter, and hypothyroidism in the offspring [2, 23, 24]. Iodine deficiency in the mother may contribute to hypothyroidism, goiter, fibrocystic breast disease, breast cancer, cognitive decline, and fibromyalgia [51]. Overall, iodine deficiency manifests in the thyroid gland as clinical or subclinical hypothyroidism, goiter, myxedema, and cretinism, or it may present as non-specific conditions such as stunted growth, mental retardation, and decreased intelligence [1, 2, 52]. Deficiencies of micronutrients such as vitamin A, selenium, iron, and zinc have also been shown to adversely affect iodine metabolism and thereby thyroid function [53]. The current iodine intake guidelines are the minimum for disease prevention, but not necessarily whole-body health. It is still unknown how much iodine is necessary for iodine sufficiency beyond prevention of goiter. The WHO established population medians do not consider iodine sufficiency of vulnerable groups such as women of reproductive age. All potential factors involved in iodine uptake and excretion must therefore be scrutinized to assess iodine sufficiency of a population.

Since an individual’s iodine status is dependent on iodine consumption and excretion, it would be ideal to use dietary intake to measure iodine consumption. Although adequate dietary iodine intake is important in human development, few studies have reported estimates of dietary exposure to iodine in the USA [54–56]. The leading sources of dietary iodine are dairy products, eggs, fish, and seaweed [57, 58]. The U.S. Department of Agriculture (USDA) proposed a percentage daily value (% DV) set at 150 μg (100% DV) for iodine among US adults to help individuals estimate the iodine content of diet [59, 60]. The % DV of iodine in commonly consumed US foods is as follows: one cup of reduced-fat milk, 56 μg iodine (37% DV); low-fat plain yogurt, 75 μg iodine (50% DV); one large egg, 24 μg iodine (16% DV); three ounces of fish sticks, 54 μg iodine (36% DV); baked cod, 99 μg iodine (66% DV) [57, 58]. Seaweed is the richest source of iodine because marine plants and animals concentrate iodine from seawater. One gram of dried or powdered seaweed, such as nori, wakame, kelp, or kombu, may contain iodine in the ranges of 16 μg (11% DV) to 2984 μg (1989% DV) [57]. Another major dietary source of iodine is iodized table, which was introduced in the 1920s and proven to be effective in combating iodine deficiency in the US salt [61]. Many salts sold in the USA follow the current recommendations for iodine fortification levels as a form of potassium iodate and potassium iodide [6, 10, 62]. Salt-iodization in the USA is voluntary and not mandatory unlike in many other countries, and consumers can still purchase either iodized or non-iodized salt. Although the Iodine Global Network estimates that the proportion of US households with access to iodized salt now exceeds 90%, data regarding actual usage is limited, and the contribution of iodized salt to the overall iodine sufficiency of the US population is uncertain [10, 62]. The Salt Institute has estimated that about 70% of the table salt in the USA is iodized, whereas table salt accounts for only 15% of total daily intake of salt [62]. Although iodized table salt is still an important source of iodine in the USA, adequate iodine intake may not be fully achieved with iodized salt only. A recent analysis of US iodized salt sales identified that only 53% of table salt sold at the retail level in the USA is iodized [63]. To make matters worse in the USA, processed foods or restaurant foods tend to use non-iodized salt due to the alleged adverse effect on the quality and taste of food [64, 65]. The USDA also does not mandate the listing of iodine content on food packaging. It is assumed that the majority of salt consumption in the USA comes from processed foods, which primarily uses non-iodized salt during production [57]. The vast majority of sodium intake is estimated to come from packaged and restaurant foods. It is estimated that only 11–12% comes from salt added to the table or salt added during home cooking. Also, the USDA food database for iodine is incomplete and reliance on dietary recall will not provide accurate results [57, 58].

A high degree of variability also exists in the iodine content of various dietary sources of iodine. The diets of cattle and chickens are often supplemented with kelp, iodine-rich seaweed, which results in variable amounts of iodine in meats, milk, and eggs [37]. Another source of iodine has been the use of iodophors in the dairy industry; iodophors are cleaning agents containing iodine used to sanitize the machinery in dairy operations and clean cow teats [66]. They are used unevenly, and some of the iodine from teat dips is absorbed and found in milk and meat [67]. There is little information whether iodophors are still widely used as there are other options for sanitizers including some that contain chlorine, an endocrine disruptor, and competitor of iodine. In the late 1950s, some bakeries were adding iodate to commercial bread mix as a dough conditioner or bread stabilizer [68], which provided a significant source of iodine in the average American diet. However, bread makers stopped using this iodide additive in the 1980s, possibly due to pressure exerted by health policymakers and the preference of brominated flour [69].

Many individuals in the USA consume restricted diets for various reasons and may have eliminated iodine-rich foods from their diets. Dairy, especially milk consumption, by adults and children, has declined in the USA. Consuming dairy products was the principal indicator of iodine status for both men and women [34, 37]. Milk has been limited or eliminated in many diets for reasons such as lactose intolerance, allergies, or believing it is a high-fat, high-calorie food. Eggs have been restricted from many people’s diets due to the high cholesterol levels in egg yolk and its relationship to heart disease. Just a few decades ago, sodium was implicated as the cause of hypertension; doctors and dietitians were instructing patients to avoid using table salt leading to the elimination of salt from the diets of many Americans. The recent change in the dietary guidelines for reduction of salt intake from 3500 to 2300 mg/day further exacerbates the issue of reduced iodine intake.

Iodine deficiency may also be caused by substances called goitrogens which inhibit or block iodine uptake into the thyroid [1, 2]. Iodine is part of the halide group along with bromine, chlorine, and fluorine. These halides act as goitrogens and compete with iodine uptake into the thyroid, especially under iodine deficiency conditions. The thyroid requires iodine to produce thyroid hormones; however, if the other halides are dominant, then iodine may not be utilized for thyroid hormone production [70]. If goitrogens subvert iodine uptake, it may be lost by excretion in the urine. It is not known if this could induce a falsely high urinary iodine excretion. Goitrogenic factors may have some role in declining iodine measurement; however, their role may be underestimated. In the USA, the impact of iodine-disrupting elements on the iodine status of the population, especially on women of reproductive age, is not known. Goitrogens found in cruciferous vegetables and lima beans are commonly consumed in the USA. Bromine as potassium bromate replaced iodate in bread in the 1970s over concern of excessive iodine consumption. Potassium bromate is a renal carcinogen and increases iodine excretion [71, 72]. Other goitrogens hurting iodine status are perchlorate, bisphenol A, oral contraceptives, amiodarone, flavonoids, and polyphenols [73]. Perchlorate has been discovered in contaminated groundwater and has also been detected in vegetables and dairy products. Perchlorate blocks iodine intake via the sodium iodide symporter (NIS), which is a membrane protein that transports iodide into the thyroid or into milk of mammals. For this reason, perchlorate has a strong goitrogenic effect [74–76]. An analysis of the NHANES 2007–2008 dataset determined that perchlorate along with thiocyanate and low iodine reduce thyroid function exhibited as decreased circulating T_4_ [77]. Another highly toxic substance affecting thyroid hormones during low iodine status is serum perfluoroalkyl acids (PFAS). These chemicals are found in stain, carpet, food packaging, paints, and stains. When iodine levels are low, and TPO antibodies are present, PFAS may disrupt thyroid function and alter thyroid hormone levels [73, 77]. Flavonoids, primarily from soy and millets, may become goitrogenic if consumed excessively. Vegetables, fruit, and supplements contain flavonoids, such as quercetin, in significant amounts. Flavonoids are healthy substances possessing antioxidant, antiviral, apoptotic, and anti-inflammatory qualities. However, excessive consumption coupled with iodine-deficient conditions may disrupt thyroid function. Similarly, resveratrol found in grapes and berries is a polyphenol with health-promoting properties, but overconsumption may inhibit the NIS gene expression thus blocking iodine uptake [78].

The American Thyroid Association (ATA) recommends that women of reproductive age, planning to become pregnant, and pregnant and lactating women consume between 220 and 290 μg/day of iodine, to compensate for fetal and maternal requirement and losses [6, 24]. Iodine levels below these guidelines may lead to iodine deficiency disorders (IDD) in the mother and offspring. Women of childbearing age, 15–44 years, are at the highest risk for iodine deficiency. A Boston study reported that 49% of pregnant and lactating women were consuming iodine below the RDA of 150 μg/day [79]. Educating women of reproductive age is crucial to create greater awareness of the importance of adherence to WHO and ATA recommendations for iodine nutrition. Iodine supplementation may need to be started well in advance of pregnancy for maximum benefit. An observational study by Moleti et al. [80] demonstrated that consumption of iodized salt for more than 2 years before pregnancy was associated with lower TSH and lower rates of gestational hypothyroidism than supplementation which commenced only in pregnancy. Prolonged use of iodized salt is associated with better maternal thyroid function, probably due to greater intra-thyroidal iodine stores to utilize during pregnancy. A further study by the same group reported higher TSH concentrations in women who took iodine supplements from early gestation compared to women who consumed iodized salt alone from 2 years before conception or those who took no supplements at all [81]. Similar comparison studies in the US population of women of reproductive age are lacking.

We have hypothesized that the lack of mandatory salt iodization programs in the USA, changes in dietary patterns leading to a reduction in iodine intake, the decline in bread fortification, increased consumption of processed foods that lack iodine, consumption of goitrogens, reduced salt guidelines, and certain geographic and socioeconomic factors may have an impact on iodine nutrition status of women of reproductive age in the USA resulting in iodine deficiency in this vulnerable population. In addition to these factors, awareness of the importance of adequate iodine nutrition, particularly during preconception, pregnancy, and lactation, among the US populace is lacking indicating cause for a significant public health concern that needs to be addressed. It is possible that all the above-discussed factors could be contributing to the reemergence of iodine deficiency in the US population, with women of reproductive age being the most vulnerable to the effects of decreased iodine intake.

### Limitations and Directions for Future Research

The strengths of this review are as follows: this scoping review synthesizes evidence on the emerging topic of iodine deficiency in women of reproductive age in the USA, the search strategy included multiple electronic bibliographic databases, the reference list of 12 different articles, internet search engines, the websites of relevant organizations, citations, and articles were reviewed by two independent reviewers who met in regular intervals to discuss results to ensure that all citations and articles were properly accounted for during the process, and an updated search was also performed in April 2018 to enhance the timeliness of this review. For this reason, this review provides a broad overview of evidence of emerging iodine deficiency among the vulnerable population subset of women of reproductive age in the USA. The limitations of this scoping review are the following: this review may have missed some relevant studies due to the English language restriction, and the selection process being limited to peer-reviewed journal articles. It is possible that searching other databases, including gray literature in the search, and the inclusion of studies published outside of the USA may have identified additional relevant studies. However, since the scope of the review was to assess the articles published in the USA and analyzing the iodine status data for the US population subset of women of reproductive age, we believe that these limitations are insignificant. Directions for future research should include conducting a more extensive systematic review and/or a meta-analysis to assess the magnitude of iodine deficiency in women of reproductive age in the USA, including studies that may have evaluated thyroid hormone status markers to assess iodine nutrition status of women of reproductive age in the USA, and conducting a more exhaustive search of gray area literature to include all possible resources of iodine nutrition in the USA.

## Conclusions

Since the first NHANES in 1974, median urinary iodine concentrations for women of reproductive age have decreased below the WHO recommendation indicating that majority of women in the childbearing years are iodine deficient. Any degree of deficiency in women may have repercussions on their health and wellbeing; however, the more significant question is what impact iodine deficiency has on her offspring. A simple, inexpensive remedy of adequate dietary iodine intake and possible iodine supplementation could be recommended to all women of reproductive age, but the concern over excess iodine outweighs the current situation. Majority of the studies included in this review demonstrate emergent iodine deficiency in this population. Iodine deficiency is a major concern and should be addressed with a sense of immediacy. Since the time of David Marine and the iodization of salt was of national prominence, our country has believed the populace was “safe” from iodine deficiency. The most recent studies would suggest otherwise and a greater emphasis must be placed on ensuring that women, especially, of childbearing ages, 18–44, receive sufficient iodine. The long-term impact of iodine deficiency in women of reproductive age in the USA cannot be overemphasized.
